# Whitening and Anti-Inflammatory Activities of Exosomes Derived from *Leuconostoc mesenteroides* subsp. DB-21 Strain Isolated from *Camellia japonica* Flower

**DOI:** 10.3390/molecules30051124

**Published:** 2025-02-28

**Authors:** Byeong-Min Choi, Gibok Lee, Hyehyun Hong, Chang-Min Park, Areum Yeom, Won-Jae Chi, Seung-Young Kim

**Affiliations:** 1 Department of Life Science and Biochemical Engineering, Sunmoon University, Asan 31460, Republic of Korea; smtm03@naver.com (B.-M.C.); kongjjo123@naver.com (H.H.); 2 R&D Center, Hankook Cosmetics Manufacturing Co., Ltd., 35 Cheonggyecheon-ro, Jongno-gu, Seoul 03188, Republic of Korea; kbok1988@hkcosm.com (G.L.); cmpark@hkcosm.com (C.-M.P.); areum0798@hkcosm.com (A.Y.); 3 Biodiversity Research Department, Species Diversity Research Division, Incheon 22689, Republic of Korea; wjchi76@korea.kr

**Keywords:** α-MSH, anti-inflammation, anti-melanogenic, B16F10 melanoma cells, MAP kinase, melanogenesis

## Abstract

In the present study, we investigated the anti-inflammatory and anti-melanogenic effects of *Leuconostoc mesenteroides* subsp. DB-21-derived exosomes (DB-21 exosomes), isolated from *Camellia japonica* flower in lipopolysaccharide (LPS)-induced RAW 264.7 macrophage cells and melanocyte-stimulating hormone (α-MSH)-induced B16F10 melanoma cells. We confirmed that DB-21 exosomes were not toxic to LPS-induced RAW 264.7 macrophage cells and α-MSH-induced B16F10 melanoma cells. Moreover, we confirmed that DB-21 exosomes inhibit the pro-inflammatory cytokines IL-6, IL-1β, TNF-α, PGE_2_, and the expression of inflammatory factors iNOS and COX-2. We also found that DB-21 exosomes have a concentration-dependent ability to inhibit melanin, TRP-1, TRP-2, tyrosinase, and MITF, which are factors involved in melanogenesis. Additionally, it inhibits the phosphorylation of Akt and GSK-3β, and MAP kinase pathway proteins such as ERK, JNK, and p38. We confirmed that DB-21 exosomes inhibit melanin synthesis in B16F10 cells through various pathways, and based on previous results, they may be used as a functional cosmetic material with anti-inflammatory and anti-melanogenic activities.

## 1. Introduction

Inflammation is a complex biological process in which the immune system responds to harmful stimuli such as pathogens, damaged cells, or irritants [1,2]. It plays a crucial role in protecting the body by eliminating harmful agents and initiating tissue repair. While this response is essential for survival, excessive or prolonged inflammation can lead to adverse effects [3,4]. For instance, chronic inflammation is a contributing factor in numerous diseases, including arthritis, cardiovascular conditions, and cancer [5,6,7]. Acute inflammation is typically characterized by redness, swelling, heat, and pain, whereas chronic inflammation often occurs due to persistent infections, autoimmune reactions, or prolonged exposure to harmful substances. As inflammation involves the recruitment of immune cells and the release of chemical mediators, its dysregulation can result in tissue damage and disease progression [8]. Hence, understanding and regulating inflammatory processes are essential for preventing inflammation-related disorders and developing therapeutic strategies.

Melanogenesis is a metabolic process by which melanin is produced in melanocytes. Melanin biosynthesis is a major physiological defense mechanism of human skin to protect against many harmful effects, such as DNA damage to skin cells by ultraviolet (UV) light [9,10]. Although melanin biosynthesis is an indispensable action to protect skin cells, excess skin pigment production causes various skin diseases such as melasma, actinic keratosis, and skin cancer [11,12]. Hyperpigmentation is related to various biological and environmental factors including inflammation and UV rays, but effective treatment for pigmentation disorders is based on inhibiting melanin production [13,14,15]. Therefore, selecting natural compounds that can regulate or inhibit melanogenesis and developing them as valuable materials for pharmaceutical and biomedical purposes is necessary.

Melanogenesis involves a serial enzymatic reaction that converts tyrosine to melanin [16]. Tyrosinase is a rate-limiting enzyme that catalyzes the oxidation of L-tyrosine to L-dihydroxyphenylalanine (L-DOPA) and further to DOPA-quinone and finally plays an important role in creating a melanin polymer [17]. Tyrosinase expression is controlled by microphthalmia-associated transcription factor (MITF), which is responsible for regulating the differentiation, proliferation, and survival of melanocytes [18,19]. A-melanocyte-stimulating hormone (α-MSH) stimulation activates the cAMP–PKA–CREB (cyclic adenosine monophosphate–protein kinase A–cAMP response element binding protein) axis and upregulates MITF transcription [20,21]. Overexpressed MITF further stimulates tyrosinase, tyrosinase-related protein 1 (TRP-1), and tyrosinase-related protein 2 (TRP-2) production [22,23]. Therefore, regulating melanogenesis signaling pathways or inhibiting tyrosinase activity may restore over-melanogenesis disorders.

Exosomes are approximately 30–150 nm vesicles of membrane structure secreted from various cells [24]. They were first discovered by Harding, Heuser, and Stahl while studying the maturation process of reticulocytes into erythrocytes [25,26]. Exosomes were initially thought to function only as cellular trash disposals because they simply removed unnecessary proteins and other molecules. However, since exosomes contain active substances such as proteins, nucleic acids, and lipids, their physiological roles have been studied in diverse fields including cancer progression, inflammation, cell proliferation, immune response, and neuronal function [27,28]. Exosomes have also been discovered in lactic acid bacteria (LAB) having immune modulation, anti-cancer, and anti-oxidant effects [29,30,31]. In addition, the extracellular vesicle of LAB has beneficial pro- and post-biotic activities on human skin conditions [32].

*Camellia japonica* is an evergreen tree belonging to the genus *Camellia* in the family Theaceae. Several studies have shown that *C. japonica* extract has many useful phytochemicals such as phenolic compounds, terpenoids, and fatty acids as well as anti-oxidant, anti-inflammatory, anti-microbial, and anti-cancer activities [33,34,35,36]. In the cosmetic field, *C. japonica* has been used as a valuable source for soothing and moisturizing skin. Particularly, *C. japonica* oil and flower extract suppresses the effect of MMP-1 induced by UVA, and has anti-melanogenic and anti-pollution effects, which causes procollagen production and skin barrier improvement [37,38,39].

In this study, we evaluated the anti-inflammation and anti-melanogenic effect of *Leuconostoc mesenteroides* subsp. DB-21 exosomes derived from *C. japonica* flowers (Lm-EVs). The Lm-EVs significantly suppress not only LPS-induced nitric oxide production in RAW 264.7 macrophage cells but also α-MSH-induced melanin synthesis in B16F10 mouse melanoma cells.

## 2. Results and Discussion

### 2.1. Exosome Analysis

**Figure 1:**
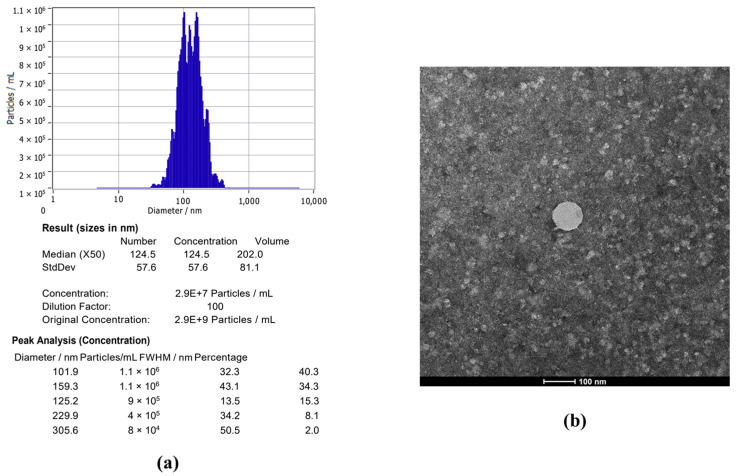
Exosome characterization of *Leuconostoc mesenteroides* subsp. DB-21-derived exosomes (DB-21 exosomes). (**a**) Nanoparticle Tracking Analysis of DB-21 exosomes. (**b**) Transmission Electron Microscopy image of DB-21 exosomes.

Nanoparticle tracking analysis (NTA) was utilized in order to assess the size and concentration of DB-21 exosomes purified via the TFF system. The results showed that a 100-fold diluted DB-21 exosome sample had a concentration of 2.9 × 10^7^ particles/mL, with the majority of particles falling within the size range of 50 to 200 nm. Furthermore, transmission electron microscopy (TEM) was employed to visualize and also confirm the morphology and size of the DB-21 exosomes (Figure 1a), successfully capturing TEM images (Figure 1b). These findings indicated that the DB-21 exosomes were highly purified, making them suitable for subsequent experiments.

### 2.2. Cytotoxicity Assessment

**Figure 2:**
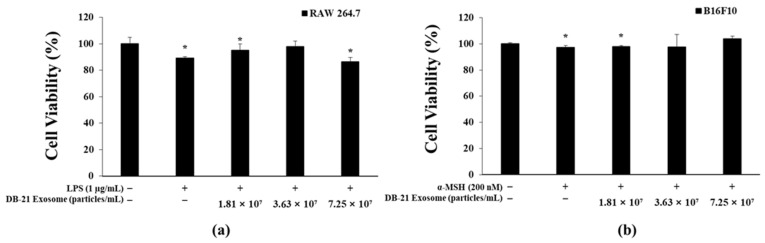
Cell viability of DB-21 exosomes on lipopolysaccharide (LPS)-stimulated RAW 264.7 cells and alpha-melanocyte-stimulating hormone (α-MSH)-induced B16F10 melanoma cells. (**a**) Cell viability of RAW 264.7 macrophage cells. (**b**) Cell viability of B16F10 melanoma cells. The cytotoxicity of RAW 264.7 cells was determined using the 3-(4,5-dimethylthiazol-2-yl)-2,5-diphenyl-2*H*-tetrazolium bromide (MTT) assay for LPS (1 μg/mL)-stimulated cells in the presence of DB-21 exosomes (1.81 × 10^7^, 3.63 × 10^7^, and 7.25 × 10^7^ particles/mL). The cytotoxicity of B16F10 cells was determined using the MTT assay for α-MSH (200 nM)-stimulated cells in the presence of DB-21 exosomes (1.81 × 10^7^, 3.63 × 10^7^, and 7.25 × 10^7^ particles/mL). Results are expressed as percentages compared with the respective values obtained for the control. * *p* < 0.05.

MTT assay utilizes the ability of mitochondria to reduce the yellow, water-soluble substrate (MTT tetrazolium) to the blue-violet, non-water-soluble (MTT formazan) (3-(4,5-dimethylthiazol-2-yl)-2,5-diphenyl-tetrazolium bromide) by dehydrogenase [40]. To investigate the effects of DB-21 exosome on B16F10 melanoma cell survival, an MTT assay was performed after co-treatment with 200 nM α-MSH and 1.81 × 10^7^, 3.63 × 10^7^, or 7.25 × 10^7^ particles/mL samples. The experiment showed that the viability of RAW 264.7 and B16F10 cells after treatment with DB-21 exosome at all concentrations was >80% (Figure 2a,b). This suggests that the samples do not exhibit toxicity to cells at the measured concentrations, and subsequently, the melanin and tyrosinase production inhibitory activities of *L. mesenteroides* subsp. DB-21-derived exosomes were assessed to determine their whitening capabilities.

### 2.3. Nitric Oxide Production Inhibitory Activity Measurement

**Figure 3:**
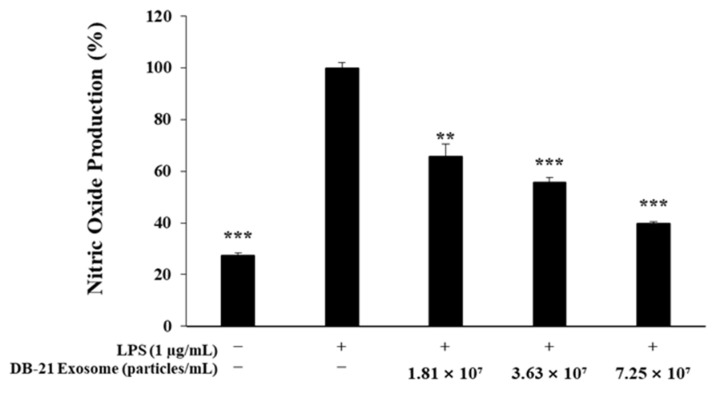
Nitric oxide production inhibition by DB-21 exosomes in LPS-stimulated RAW 264.7 cells. The production of nitric oxide in LPS (1 µg/mL)-stimulated RAW 264.7 cells in the presence of DB-21 exosomes (1.81 × 10^7^, 3.63 × 10^7^, and 7.25 × 10^7^ particles/mL). The results are expressed as percentages compared with the respective values obtained for the control. ** *p* < 0.01; *** *p* < 0.001.

In this study, the anti-inflammatory activity of DB-21 exosomes was assessed by treating RAW 264.7 macrophages with LPS and DB-21 exosomes, followed by measuring the amount of nitric oxide production. These results demonstrated that DB-21 exosomes inhibited nitric oxide production in a concentration-dependent manner. Notably, at the highest concentration of 7.25 × 10^7^ particles/mL, nitric oxide production was suppressed to levels comparable to those of the LPS-untreated control group (Figure 3). Based on these findings, subsequent experiments were conducted to investigate the expression of the pro-inflammatory cytokines involved in NO synthesis.

### 2.4. Prostaglandin E2 Production Inhibitory Activity Measurement

**Figure 4:**
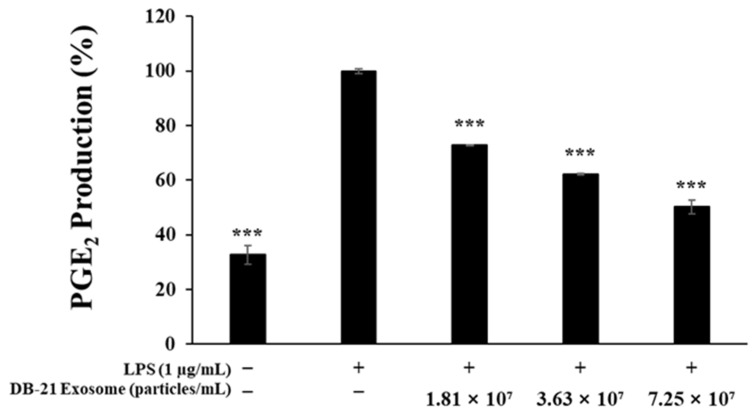
Inhibitory effects of DB-21 exosomes on prostaglandin E_2_ (PGE_2_) production in LPS-stimulated RAW 264.7 cells. PGE_2_ production in LPS (1 μg/mL)-stimulated RAW 264.7 cells in the presence of DB-21 exosomes (1.81 × 10^7^, 3.63 × 10^7^, and 7.25 × 10^7^ particles/mL). Results are expressed as percentages compared with the respective values obtained for the control. *** *p* < 0.001.

Prostaglandins are produced through the conversion of arachidonic acid by cyclooxygenase, with prostaglandin E2 (PGE_2_) known to suppress the functions of lymphocytes and macrophages via the prostaglandin E receptor (EP) [41]. To evaluate whether the DB-21 exosomes could inhibit PGE_2_ activity, an experiment was conducted, that revealed that DB-21 exosomes reduced LPS-induced PGE_2_ activity in a concentration-dependent manner at concentrations of 1.81 × 10^7^, 3.63 × 10^7^, or 7.25 × 10^7^ particles/mL (Figure 4).

### 2.5. Pro-Inflammatory Cytokine (IL-6, IL-1β, TNF-α) Production Inhibitory Activity Measurement

**Figure 5:**
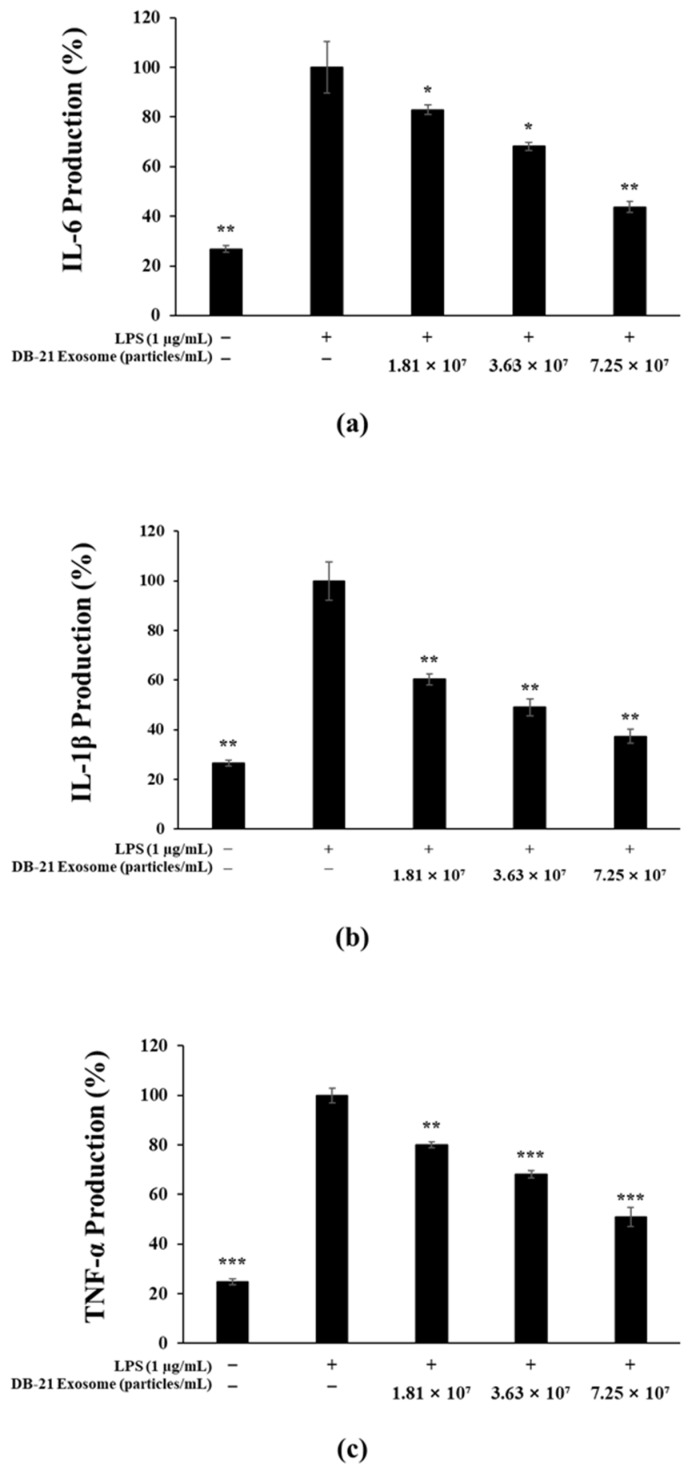
Inhibition of pro-inflammatory cytokines by DB-21 exosomes in LPS-stimulated RAW 264.7 cells. Production of (**a**) interleukin (IL)-6, (**b**) IL-1β, and (**c**) tumor necrosis factor-alpha (TNF-α) in LPS (1 μg/mL)-stimulated RAW 264.7 cells in the presence of DB-21 exosomes (1.81 × 10^7^, 3.63 × 10^7^, and 7.25 × 10^7^). Results are expressed as percentages compared with the respective values obtained for the control. * *p* < 0.05; ** *p* < 0.01; *** *p* < 0.001.

The upregulation of pro-inflammatory cytokines promotes the production of reactive nitrogen species (RNS), including nitric oxide, which plays a key role in the development of inflammatory diseases [42,43]. Accordingly, we investigated the inhibitory effects of DB-21 exosomes on pro-inflammatory cytokines. The findings revealed that DB-21 exosomes significantly suppressed the expression of IL-6, IL-1β, and TNF-α in a concentration-dependent manner (Figure 5a–c). Furthermore, the performed western blot analysis confirmed the modulation of iNOS and COX-2 expression, which are key mediators involved in the inflammatory response.

### 2.6. Melanin Production Inhibition Activity Assessment

**Figure 6:**
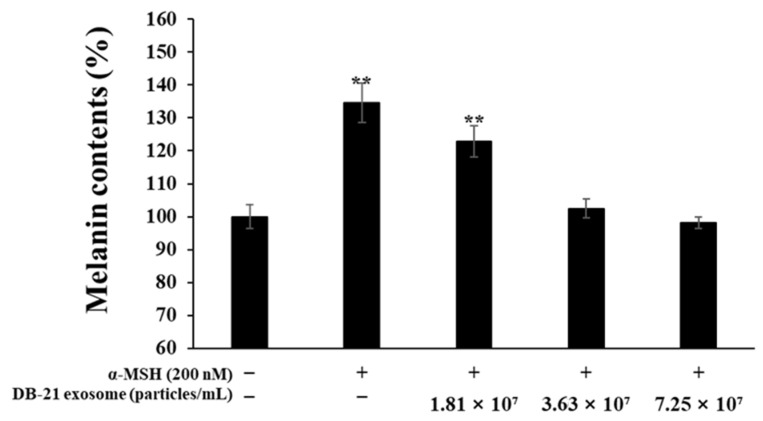
Effect of DB-21 exosomes on melanin synthesis in B16F10 melanoma cells. The production of melanin was assayed in the cell pellets of α-MSH (200 nM)-stimulated cells for 72 h in the presence of DB-21 exosomes (1.81 × 10^7^, 3.63 × 10^7^, and 7.25 × 10^7^ particles/mL). Data represent the means ± standard deviation (SD) with three separate experiments. ** *p* < 0.01.

In this experiment, the melanin production from α-MSH-stimulated B16F10 melanoma cells was measured to investigate the melanin production inhibitory activity of DB-21 exosome in the presence of 200 nM α-MSH and co-treatment with 1.81 × 10^7^, 3.63 × 10^7^, or 7.25 × 10^7^ particles/mL samples. The 7.25 × 10^7^ DB-21 exosome-treated group exhibited a concentration-dependent increase in melanin inhibitory activity, with similar inhibitory activity to the α-MSH-untreated group. It is suggesting that DB-21 exosomes are effective in inhibiting melanin production (Figure 6). To further determine the value of each sample as an ingredient with whitening activity, its inhibitory activity against tyrosinase, an important rate-limiting enzyme in the early stages of melanin synthesis, was assessed.

### 2.7. Tyrosinase Production Inhibition Activity Assessment

**Figure 7:**
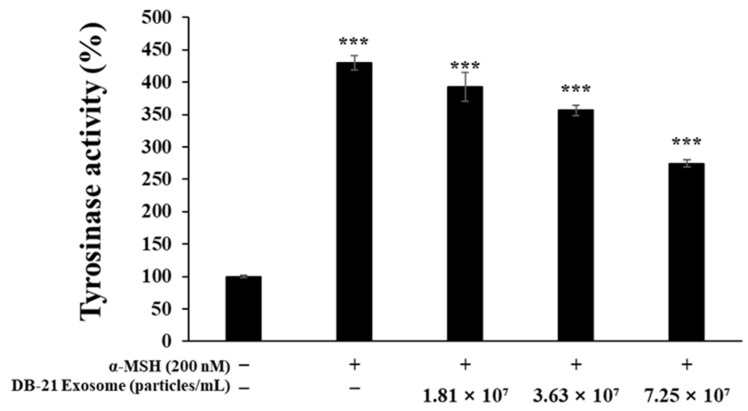
Effect of DB-21 exosomes on tyrosinase activity in B16F10 melanoma cells. The cells were stimulated with α-MSH (200 nM for 72 h in the presence of DB-21 exosomes (1.81 × 10^7^, 3.63 × 10^7^, and 7.25 × 10^7^ particles/mL). The effect of DB-21 exosomes on tyrosinase activity was determined by measuring the absorbance at 490 nm. The results are expressed as a percentage of the control. Data represent the means ± standard deviation (SD) with three independent experiments. *** *p* < 0.001.

Tyrosinase is a key enzyme in the melanin synthesis step, oxidizing tyrosine to DOPA-quinone ultimately contributes to melanin synthesis. Therefore, tyrosinase production inhibition directly decreases melanin synthesis [44,45,46]. In this experiment, to investigate the effects of DB-21 exosome on tyrosinase activity in B16F10 melanoma cells, the cells were incubated with 200 nM α-MSH and 1.81 × 10^7^, 3.63 × 10^7^, or 7.25 × 10^7^ particles/mL DB-21 exosomes, and the intracellular proteins were extracted to measure the tyrosinase production. The results showed a concentration-dependent decrease in tyrosinase production in the DB-21 exosome treatment group (Figure 7). Based on the results of melanin and tyrosinase production inhibition activity assessments, DB-21 exosome has significant whitening activity; therefore, further experiments were conducted using DB-21 exosome with whitening activity.

### 2.8. Western Blot Analysis

**Figure 8:**
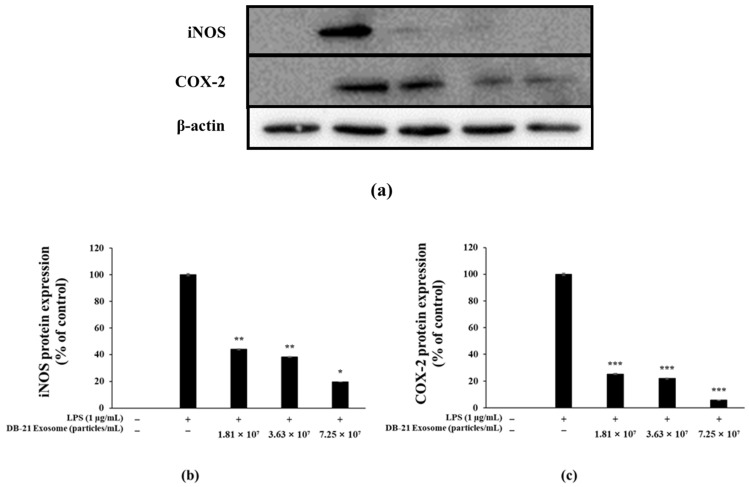
Inhibitory effects of DB-21 exosomes on inducible nitric oxide synthase (iNOS) and cyclooxygenase 2 (COX-2) protein expression in LPS-stimulated RAW 264.7 cells. Inhibitory effect of DB-21 exosome on the protein levels of (**a**) The protein band detection results, (**b**) iNOS, and (**c**) COX-2 in RAW 264.7 cells stimulated with LPS (1 μg/mL) in the presence of DB-21 exosomes (1.81 × 10^7^, 3.63 × 10^7^, and 7.25 × 10^7^ particles/mL). Expression of iNOS, COX-2, and β-actin were determined by western blotting. Data represent the means ± standard deviation (SD) with three independent experiments. * *p* < 0.05; ** *p* < 0.01; *** *p* < 0.001.

**Figure 9:**
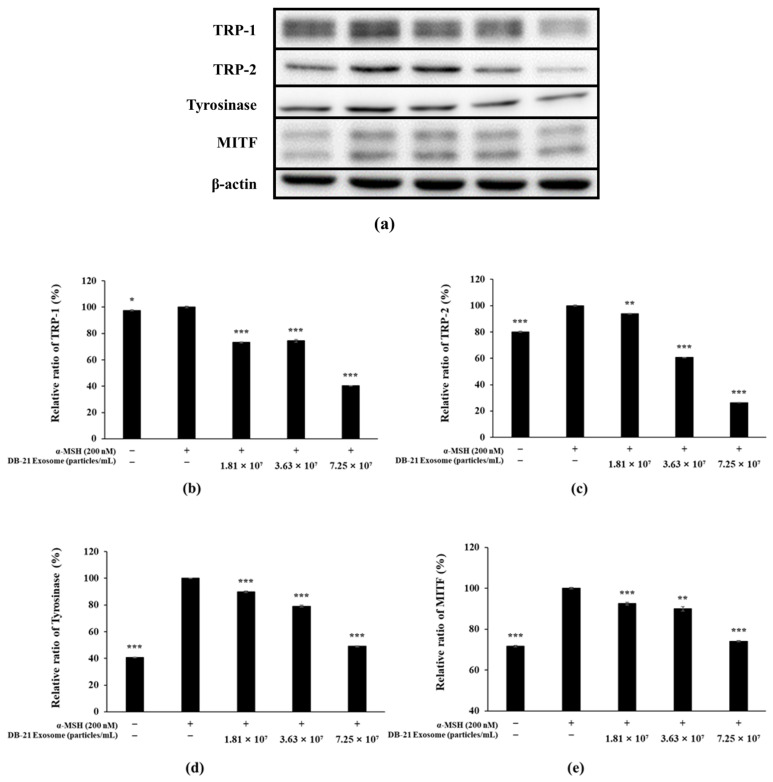
Western blot analysis of Tyrosinase-related protein (TRP)-1, TRP-2, Tyrosinase, and microphthalmia-associated transcription factor (MITF) in α-MSH-induced B16F10 melanoma cells. Cells were pre-incubated for 48 h, and then treated with α-MSH (200 nM) and DB-21 exosomes (1.81 × 10^7^, 3.63 × 10^7^, and 7.25 × 10^7^ particles/mL). (**a**) The protein band detection results, (**b**) Tyrosinase-related protein (TRP)-1, (**c**) TRP-2, (**d**) Tyrosinase, and (**e**) MITF protein levels were analyzed using western blotting. β-actin was used as the control. Data represent the means ± standard deviation (SD) with three independent experiments. * *p* < 0.05; ** *p* < 0.01; *** *p* < 0.001.

PGE_2_ activity is regulated by COX-2 expression, which ultimately induces inflammatory responses [47]. Furthermore, iNOS catalyzes the production of nitric oxide by oxidizing l-arginine, indicating that inhibition of both iNOS and COX-2 can effectively modulate inflammatory processes [48]. Melanin is synthesized by tyrosinase through the oxidation of L-tyrosine to L-DOPA, which in turn is oxidized to DOPA-quinone, which is synthesized under the influence of TRP-1 and TRP-2 [49,50]. Microphthalmia-associated transcription factor (MITF) is deeply involved in these processes. In this experiment, western blots were performed to determine the effect of DB-21 exosomes on iNOS, COX-2, TRP-1, TRP-2, tyrosinase, and MITF expression in LPS-induced RAW 264.7 macrophage cells and α-MSH-stimulated B16F10 melanoma cells. Our results showed that DB-21 exosomes effectively inhibited iNOS and COX-2 expression in LPS-induced RAW 264.7 macrophage cells (Figure 8). Additionally, TRP-1, TRP-2, tyrosinase, and MITF expression was inhibited in α-MSH-stimulated B16F10 melanoma cells (Figure 9). These findings suggest that DB-21 exosomes could serve as functional ingredients with anti-inflammatory and skin-whitening properties by inhibiting proteins directly involved in melanin synthesis. Subsequently, the effects of DB-21 exosomes on the phosphorylation levels of the MAP and Akt pathways and the upstream mechanisms of MITF were investigated.

### 2.9. MAP Kinase Phosphorylation Inhibition in DB-21 Exosomes

**Figure 10:**
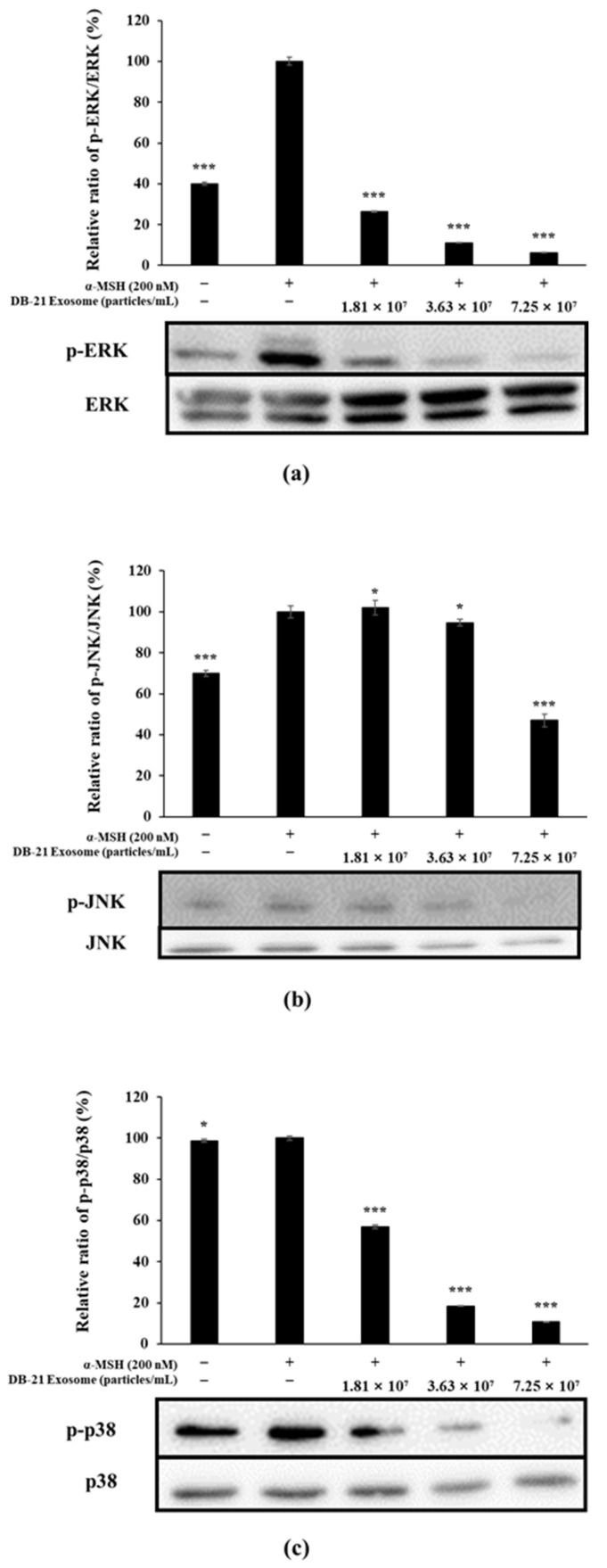
Effect of DB-21 exosomes on MAPK phosphorylation in B16F10 melanoma cells. Cells were pre-incubated for 48 h, and then treated with α-MSH (200 nM) and DB-21 exosomes (1.81 × 10^7^, 3.63 × 10^7^, and 7.25 × 10^7^ particles/mL). (**a**) analysis of phospho (p)-ERK/ERK, (**b**) p-JNK/JNK, (**c**) p-p38/p38. Protein levels were analyzed using western blotting. Data represent the means ± standard deviation (SD) with three independent experiments. * *p* < 0.05; *** *p* < 0.001.

Tyrosinase, TRP-1, and TRP-2 are directly involved in melanin production and regulated by MITF, which is stimulated by several pathways, including phosphorylation of MAP kinases such as ERK, JNK, and p38 [51,52]. In this experiment, the phosphorylation level of MAP kinase was examined by western blot. The experimental results confirmed that DB-21 exosomes effectively inhibited the phosphorylation of ERK, JNK, and p38, which belong to the MAP kinase pathway; in particular, the phosphorylation of ERK, JNK, and p38 of the highest concentration-treated group was lower than that of the α-MSH-free group. These experimental results confirmed that DB-21 exosomes inhibit the expression of MITF through the inhibition of phosphorylation of ERK, JNK, and p-38, which belong to the MAP kinase pathway, thereby inhibiting melanin production (Figure 10).

### 2.10. Akt and GSK-3β Phosphorylation Inhibition by DB-21 Exosomes

**Figure 11:**
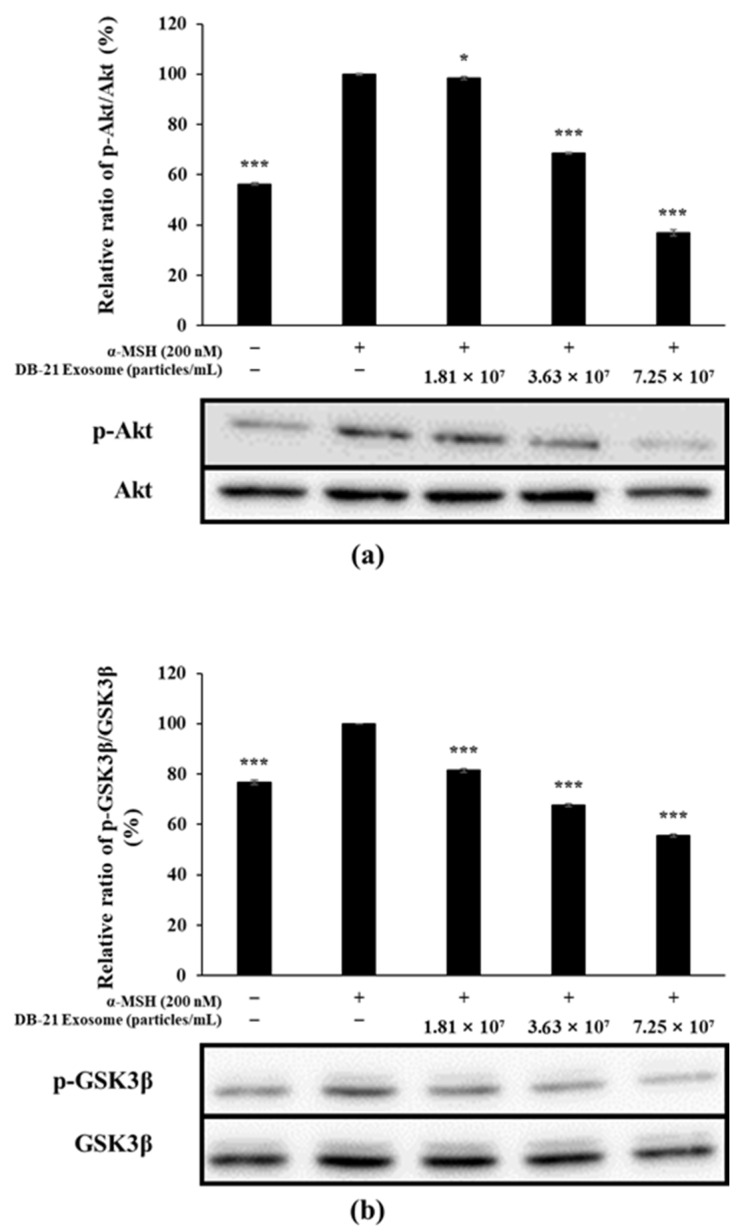
Effect of DB-21 exosomes on Akt phosphorylation in B16F10 melanoma cells. Cells were pre-incubated for 48 h, and then treated with α-MSH (200 nM) and DB-21 exosomes (1.81 × 10^7^, 3.63 × 10^7^, and 7.25 × 10^7^ particles/mL). (**a**) Analysis for p-Akt/Akt, (**b**) p-glycogen synthase kinase (GSK)-3β/GSK-3β. Protein levels were analyzed using western blotting. Data represent the means ± standard deviation (SD) with three independent experiments. * *p* < 0.05; *** *p* < 0.001.

The expression of MITF, which is directly involved in melanin production, is influenced mainly by three factors and regulated primarily by the phosphorylation of MAP kinase, Akt, and CREB [53,54]. To determine the effect of DB-21 exosomes on the level of Akt phosphorylation, western blot was performed, and the results showed that DB-21 exosomes effectively inhibited the phosphorylation of not only Akt but also GSK-3β, a downstream mechanism of Akt (Figure 11). Along with previous findings, these results confirm that the MITF-inhibitory activity of DB-21 exosomes depends on not only MAP kinase phosphorylation but also Akt and GSK-3β phosphorylation. This suggests that the whitening activity of DB-21 exosomes is not mediated through a single pathway, but at least two signaling pathways. These results demonstrate the potential of DB-21 exosomes as a material with a whitening function, and further studies should investigate the bioactivity of DB-21 exosomes.

## 3. Materials and Methods

### 3.1. Incubation of Leuconostoc mesenteroides subsp. DB-21

The *L. mesenteroides* subsp. DB-21 strain used in this experiment was isolated from *C. japonica* flowers collected from Bulgap Mountain, Moak-ri, Bulgap-myeon, Younggwang-gun, and Jeollanam-do regions, and provided by the National Institute of Biological Resources (NIBR, Incheon, Republic of Korea) on 22 March 2019 [55].

### 3.2. Exosome Isolation

*L. mesenteroides* subsp. DB-21 strain was cultured using de Man, Rogosa, and Sharpe (MRS) broth at 30 °C and 200× *g* for 18 h and then centrifuged at 4000× *g* for 15 min to recover the culture supernatant. The collected supernatant was filtered using a 0.22 μm pressure-sensitive filter (SPL, Pocheon, Republic of Korea). The exosomes were subsequently purified using an Äkta flux S (Cytiva, Amersham, UK) instrument with a tangential flow filtration (TFF) system, where a hollow fiber cartridge with 0.5 mm fiber ID, 110 cm flow path, and 100,000 nanomolecular weight cut-off (NMWC) pore size was used as the TFF filter.

### 3.3. Characterization of L. mesenteroides subsp. DB-21 Derived Exosomes

Exosome analysis was carried out using nanoparticle tracking analysis (NTA) and transmission electron microscopy (TEM). NTA measurements were taken with a ZetaView PMX 110 (Particle Metrix, Meerbusch, Germany) and processed using ZetaView software (version 8.05.16 SP3). For TEM, an Alos L120C (FEI, Hillsboro, OR, USA) was used, where exosome samples were applied to a formvar-coated copper grid, stained with 2% uranyl acetate for 20 s, and then blotted.

### 3.4. Materials and Cell Culture

The α-melanocyte-stimulating hormone (α-MSH), lipopolysaccharide (LPS), Griess reagent, and L-DOPA were purchased from Sigma-Aldrich (St. Louis, MO, USA), and B16F10 melanoma and RAW 264.7 cells were obtained from American type cell culture (ATCC, Manassas, VA, USA). Dulbecco’s Modified Eagle’s Medium (DMEM, Welgene, Gyeongsan, Republic of Korea), containing 10% fetal bovine serum (FBS, Welgene, Gyeongsan, Republic of Korea), 100 µg/mL penicillin, and 100 µg/mL streptomycin, was used to culture the cells. RAW 264.7 and B16F10 cells were stabilized by passaging in an incubator under humid conditions with 5% CO_2_ at 37 °C for 2 days and 3 days, respectively.

### 3.5. Cytotoxicity Measurement

To determine the effect of *L. mesenteroides*-derived exosomes (DB-21 exosomes) on the survival of LPS-induced RAW 264.7 cells and α-MSH-induced B16F10 melanoma cells, 8.0 × 10^4^ RAW 264.7 cells/well were seeded in 24-well plates and pre-incubated for 24 h in a 37 °C, 5% CO_2_ incubator, followed by co-treatment with 1 μg/mL LPS and DB-21 exosomes (1.81 × 10^7^, 3.63 × 10^7^, and 7.25 × 10^7^ particles/mL) for 24 h. Similarly, 1.0 × 10^4^ B16F10 melanoma cells/well were seeded in 24-well plates and pre-incubated for 24 h in a 37 °C, 5% CO_2_ incubator, followed by co-treatment with 200 nM α-MSH and DB-21 exosomes (1.81 × 10^7^, 3.63 × 10^7^, and 7.25 × 10^7^ particles/mL) for 72 h. Subsequently, 3-(4,5-dimethylthiazol-2-yl)-2,5-diphenyltetrazolium bromide (MTT, Sigma-Aldrich) reagent was added to each well and incubated for 3 h at 37 °C, 5% CO_2_ incubator to form formazan blue. Formazan blue was then dissolved by adding dimethyl sulfoxide (DMSO, Sigma-Aldrich) to each well after removing the supernatant, then transferred to a 96-well plate to measure the absorbance at 570 nm using a microplate reader (Thermo Fisher Scientific, Waltham, MA, USA).

### 3.6. Nitric Oxide Inhibition Activity Measurement

To investigate the nitric oxide (NO) inhibitory activity of DB-21 exosome, 8.0 × 10^4^ RAW 264.7 cells/well were seeded in a 24-well plate, incubated in a 37 °C, 5% CO_2_ incubator for 24 h, and then simultaneously treated with 1 μg/mL LPS and 1.81 × 10^7^, 3.63 × 10^7^, or 7.25 × 10^7^ DB-21 exosome particles/mL. After 24 h, 100 μL culture medium and Griess reagent [1% (*w*/*v*) sulfanilamide, 0.1% (*w*/*v*) naphylethylenediamine in 2.5% (*v*/*v*) phosphoric acid] were mixed equimolarly, incubated in dark for 10 min, and the absorbance at 540 nm was measured.

### 3.7. Prostaglandin E2 Production Inhibition Activity Measurement

To measure the prostaglandin E2 (PGE_2_) production inhibitory activity of DB-21 exosomes in RAW 264.7 cells, 8.0 × 10^4^ RAW 264.7 cells/well were seeded in 24-well plates and pre-incubated for 24 h in a 37 °C, 5% CO_2_ incubator, followed by simultaneous treatment with 1 μg/mL LPS and 1.81 × 10^7^, 3.63 × 10^7^, or 7.25 × 10^7^ DB-21 exosome particles/mL for 24 h. The culture medium was then collected and centrifuged at 10,000× *g* for 3 min to remove the precipitate, and the PGE_2_ amount present in the supernatant was measured using a mouse enzyme-linked immunosorbent assay (ELISA) kit (R&D Systems Inc., Minneapolis, MN, USA).

### 3.8. Pro-Inflammatory Cytokine (TNF-α, IL-6, IL-1β) Production Inhibitory Activity 

RAW 264.7 cells (8.0 × 10^4^ cells/well) were seeded in 24-well plates and cultured in a 37 °C, 5% CO_2_ incubator for 24 h, followed by co-treatment with 1 μg/mL LPS and 1.81 × 10^7^, 3.63 × 10^7^, or 7.25 × 10^7^ DB-21 exosome particles/mL for 24 h. The cell cultures were then centrifuged at 10,000× *g* for 3 min to remove the precipitate and pro-inflammatory cytokine (IL-6, IL-1β, and TNF-α) amount in the supernatant was measured using the Mouse TNF-α ELISA Kit (Invitrogen, Carlsbad, CA, USA), Mouse IL-6 ELISA Kit (BD Biosciences, San Jose, CA, USA), and Mouse IL-1β ELISA Kit (R&D Systems Inc.).

### 3.9. Melanin Content Measurement

To determine the effect of DB-21 exosomes on melanin synthesis in α-MSH-induced B16F10 melanoma cells, melanin contents were analyzed. B16F10 melanoma cells (4.0 × 10^4^ cells/well) were seeded in a 6-well plate and incubated in a 37 °C, 5% CO_2_ incubator for 24 h, followed by simultaneous treatment with 200 nM α-MSH and 1.81 × 10^7^, 3.63 × 10^7^, or 7.25 × 10^7^ DB-21 exosome particles/mL for 72 h. The cells were then detached from the plate by treatment with trypsin and lysed for 60 min in radioimmunoprecipitation assay buffer (RIPA buffer; Biosesang, Seongnam, Republic of Korea) supplemented with 1 mM Na_3_VO_4_, 1 mM PMSF, and 1% protease inhibitor. The supernatant was then removed by centrifugation (4 °C, 13,000× *g*) for 30 min, and the pellet was heated at 90 °C for 1 h with 500 µL 1 N NaOH and transferred to a 96-well plate, followed by absorbance measurement at 405 nm.

### 3.10. Tyrosinase Activity Measurements

To investigate the effect of DB-21 exosomes on the activity of tyrosinase in α-MSH-induced B16F10 melanoma cells, 4.0 × 10^4^ B16F10 melanoma cells/well were seeded in a 6-well plate, pre-incubated in a 37 °C, 5% CO_2_ incubator for 24 h, and then co-treated with 200 nM α-MSH and 1.81× 10^7^, 3.63 × 10^7^, or 7.25 × 10^7^ DB-21 exosome particles/mL for 72 h. The cells were then detached from the plate by treatment with trypsin and lysed for 60 min in RIPA buffer supplemented with 1 mM Na_3_VO_4_, 1 mM PMSF, and 1% protease inhibitor. After lysis, the supernatant was recovered by centrifugation (4 °C, 13,000× *g*) for 30 min and the protein content in the supernatant was quantified using BCA kit (Bio-Rad, Hercules, CA, USA). Subsequently, 20 µL protein quantified using BCA kit was incubated with 80 µL 2 mg/mL L-DOPA in dark for 2 h and the absorbance at 490 nm was measured.

### 3.11. Western Blotting

RAW 264.7 cells (4 × 10^5^ cells/well) were seeded in 6-well plates and incubated in a 37 °C, 5% CO_2_ incubator for 24 h, followed by co-treatment with 1 μg/mL LPS and 1.81 × 10^7^, 3.63 × 10^7^, or 7.25 × 10^7^ DB-21 exosome particles/mL for 24 h. Similarly, B16F10 melanoma cells (4 × 10^4^ cells/well) were seeded in a 6-well plate and pre-incubated for 48 h in a 37 °C, 5% CO_2_ incubator, followed by simultaneous treatment with 200 nM α-MSH and 1.78 × 10^9^, 3.55 × 10^9^, or 7.10 × 10^9^ DB-21 exosome particles/mL for 24 h. The cells were then lysed by adding RIPA buffer supplemented with 1 mM Na_3_VO_4_, 1 mM PMSF, and 1% protease inhibitor for 60 min, followed by centrifugation (4 °C, 13,000× *g*) for 30 min to separate the supernatant. The protein content of the separated supernatant was quantified using BCA kit (Bio-Rad, Hercules, CA, USA), and 20 µg quantified protein was electrophoresed using 10% sodium dodecyl sulfate-polyacrylamide gel electrophoresis (SDS–PAGE). After 60 min, the proteins were transferred to a polyvinylidene difluoride (PVDF) membrane (Millipore, Burlington, MA, USA) and blocked in 5% skim milk dissolved in 1× TBST (Tris-buffered saline, 0.1% Tween 20) for 2 h at room temperature. The membrane was then washed thrice at 10 min intervals using TBST and incubated with the primary antibodies against iNOS (1:1000, Bio-Rad), COX-2 (1:1000, Rockland Immunochemicals, Inc., Pottstown, PA, USA), MITF (1:500, Cell Signaling Technology, Danvers, MA, USA), TRP-1 (1:5000, Santa Cruz Biotechnology, Dallus, TX, USA), TRP-2 (1:500, Santa Cruz), tyrosinase (1:500, Santa Cruz), β-actin (1:5000, Bio-Rad), phospho-p44/42 MAPK (Erk1/2; Thr202/Tyr204; 1:500, Cell Signaling), phospho-SAPK/JNK (1:500, Cell Signaling), phospho-p38 MAPK (Thr180/Tyr182; 1:500, Cell Signaling), phospho-AKT (1:500, Cell Signaling), phospho-GSK-3β (Ser9, 5B3; 1:500, Cell Signaling), p44/42 MAPK Erk1/2 (1:500, Cell Signaling), SAPK/JNK (1:500, Cell Signaling), p38 MAPK (1:500, Cell Signaling), AKT (1:500, Cell Signaling), and GSK-3β (27C10; 1:500, Cell Signaling) for 18 h at 4 °C. After the reaction was completed, the membrane was washed thrice with TBST, and then incubated with the anti-mouse IgG HRP and anti-rabbit IgG secondary antibodies (Jackson ImmunoResearch, West Grove, PA, USA) diluted at 1:10,000 for 2 h at room temperature. After 2 h, the membrane was washed thrice with TBST, reacted with ECL kit (Bio-Rad, Hercules, CA, USA), and developed with imaging densitometer (model GS-700, Bio-Rad, Hercules, CA, USA). The expression of the developed proteins was quantified using ImageJ 1.52v (NIH, Bethesda, MD, USA) and then graphed.

### 3.12. Statistical Analysis

All experiments were performed in triplicate, with the results expressed as mean values ± standard deviation. Statistical analysis was performed by analysis of variance (ANOVA) followed by Student’s *t*-test for multiple comparisons to test the significance of each treatment group (* *p* < 0.05; ** *p* < 0.01; *** *p* < 0.001).

## 4. Conclusions

In the present study, we explored the anti-inflammatory and whitening properties of exosomes derived from *Leuconostoc mesenteroides* subsp. DB-21, a lactic acid bacterium (LAB) isolated from the flowers of *Camellia japonica*, a plant native to Korea. Exosomes from DB-21 were analyzed for their potential therapeutic effects, and the experimental findings revealed their significant ability to mitigate inflammatory responses and regulate melanin production.

The anti-inflammatory activity of DB-21 exosomes was demonstrated through their ability to suppress the production of key inflammatory mediators and cytokines. Specifically, these exosomes significantly reduced nitric oxide (NO) and prostaglandin E2 (PGE_2_) levels, which are critical markers of inflammation. Moreover, they effectively inhibited the secretion of pro-inflammatory cytokines, including tumor necrosis factor-alpha (TNF-α), interleukin-6 (IL-6), and interleukin-1 beta (IL-1β). These cytokines play central roles in propagating inflammatory responses. At a molecular level, DB-21 exosomes were found to downregulate the expression of inducible nitric oxide synthase (iNOS) and cyclooxygenase-2 (COX-2), both of which are pivotal enzymes involved in inflammatory pathways. Importantly, these anti-inflammatory effects were achieved at concentrations of DB-21 exosomes that showed no cytotoxic effects, as evidenced by the absence of adverse outcomes in RAW 264.7 macrophage cells. This highlights the biocompatibility of the exosomes and their potential for safe therapeutic application.

In addition to their robust anti-inflammatory properties, DB-21 exosomes demonstrated potent whitening effects by interfering with key molecular pathways involved in melanogenesis. Melanin synthesis, primarily regulated by enzymes such as tyrosinase, tyrosinase-related protein 1 (TRP-1), and tyrosinase-related protein 2 (TRP-2), as well as the microphthalmia-associated transcription factor (MITF), was significantly suppressed upon treatment with DB-21 exosomes. This suggests that the exosomes effectively target the core regulatory mechanisms of pigmentation. Further analysis revealed that DB-21 exosomes inhibited the activation of mitogen-activated protein kinase (MAPK) pathways, specifically extracellular signal-regulated kinase (ERK), c-Jun N-terminal kinase (JNK), and p38 MAPK. These pathways are known to upregulate MITF expression, thereby promoting melanin production. By disrupting these signaling pathways, the exosomes effectively suppressed melanin synthesis.

Moreover, DB-21 exosomes were observed to modulate other signaling molecules associated with pigmentation control. Specifically, they inhibited the phosphorylation of Akt and glycogen synthase kinase-3 beta (GSK-3β), both of which are linked to the regulation of melanogenesis. These findings underscore the comprehensive action of DB-21 exosomes in targeting multiple points of the melanogenesis pathway. These findings suggest that DB-21 exosomes interfere with α-melanocyte-stimulating hormone (α-MSH)-induced MITF expression by modulating the MAPK and Akt signaling pathways. By downregulating the expression of MITF, a master regulator of tyrosinase, TRP-1, and TRP-2, DB-21 exosomes effectively reduced tyrosinase activity, which directly influenced melanin synthesis. This dual action of inhibiting melanin production at both the enzymatic and transcriptional levels underscores its potential as a multifunctional whitening agent.

Based on these results, DB-21 exosomes hold promise as an active ingredient for whitening cosmetic formulations with additional anti-inflammatory benefits. However, further studies are warranted in order to identify the specific bioactive components of DB-21 exosomes responsible for these effects. Therefore, we are planning a study to identify active components from the constituent elements of exosomes, such as nucleic acids, proteins, and lipids, and we will conduct further validation experiments. Additionally, it is believed that further research should be conducted to enhance the efficacy of exosomes through changes in culture conditions and additional purification of the exosomes. Such studies may pave the way for the development of novel cosmetic and therapeutic applications by exploiting the unique properties of LAB-derived exosomes.

## Data Availability

The original contributions presented in the study are included in the article.
